# Implementation barriers and facilitators of a virtual telehealth program for patients with persistently poorly controlled type 2 diabetes mellitus: a rapid qualitative analysis approach

**DOI:** 10.1186/s12913-026-14680-2

**Published:** 2026-06-27

**Authors:** Connor D. Drake, Tiera Lanford-Davey, Madeleine R. Eldridge, Zoe E. Waddell, Jashalynn German, Abigail Shapiro, Lucy Esteve, Priyanka Tejwani, Allison A. Lewinski, Hayden B. Bosworth, David Edelman, Karen Steinhauser, Leah L. Zullig, Matthew J. Crowley

**Affiliations:** 1 Durham Veterans Affairs Center of Innovation to Accelerate Discovery and Practice Transformation (ADAPT), 411 West Chapel Hill Street, Durham, NC 27701 USA; 2 Department of Population Health Sciences, Duke University School of Medicine, Durham, USA; 3 Department of Medicine, Duke University, Durham, USA; 4 Duke University School of Nursing, Durham, USA

**Keywords:** Persistently poorly controlled type 2 diabetes, Telehealth, Virtual care, Implementation science, Qualitative research, Diabetes mellitus, Telehealth, Implementation science, Self-management

## Abstract

**Background:**

Persistently poorly controlled type 2 diabetes mellitus (PPDM) affects 10–15% of patients with diabetes and disproportionately contributes to morbidity, mortality, and expenditure. Unresponsive to standard outpatient treatment, PPDM requires intensified and comprehensive approaches that lend themselves to a telehealth delivery modality. Despite the recognized need for these types of comprehensive telehealth care models, little is known about factors that influence their implementation in real-world care settings. The objective of this study was to use a secondary qualitative analysis approach to understand implementation barriers and facilitators of a comprehensive virtual care program that has demonstrated superiority in comparison to standard telehealth for patients with PPDM.

**Methods:**

Patients (*n* = 19) and clinical and administrative staff (*n* = 8) participated in interviews to discuss their first-hand experience with implementation of comprehensive telehealth as part of a comparative effectiveness randomized controlled trial. Using the Health Equity Implementation Framework, we conducted a secondary analysis using rapid qualitative methods to identify factors that may influence the implementation of this approach into routine care delivery.

**Results:**

The comprehensive diabetes telehealth intervention complemented primary care goals to support diabetes self-management but required novel, complex patterns of communication with the existing care team. The approach was perceived as effective, but the intensity of the program introduced challenges with maintaining ongoing patient engagement. Adaptations related to standardizing provider training and integrating locally available resources and complementary programs may amplify the benefit of the approach to patients. Staffing the approach may require new clinical roles or substituting existing responsibilities to ensure sufficient capacity to deliver the approach with fidelity.

**Conclusion:**

Comprehensive diabetes telehealth intervention implementation is facilitated by using existing infrastructure and care team organization. Opportunities exist to improve fit of the program as part of routine delivery at scale. Findings have implications for the design of strategies to improve implementation efforts required to offer the program at scale to patients with PPDM.

**Clinical trial number:**

Not applicable.

## Introduction

Persistently poorly controlled type 2 diabetes mellitus (PPDM) affects 10–15% of patients with type 2 diabetes and disproportionately contributes to morbidity, mortality, and expenditure within health care systems [1, 2]. Classified as consistently elevated hemoglobin A1c (HbA1c) ≥ 8.5% for at least one year despite clinic-based management, PPDM is defined by its incomplete responsiveness to standard outpatient treatment approaches. PPDM may result from outpatient care not adequately addressing underlying medical, social, and behavioral drivers, including medication underuse, suboptimal diet/activity, and comorbid depression, resulting in ineffective glycemic control and vascular complications [1, 3]. For these reasons, patients with PPDM require intensified and innovative diabetes management programs [4, 5].

PPDM is notably high among the United States (US) Veteran population, affecting nearly 12% of 5.75 million Veterans with diabetes according to the Veterans Health Administration (VHA), the largest public integrated health system in the US [6]. Practical Telemedicine to Improve Control and Engagement for Veterans with Clinic-Refractory Diabetes Mellitus was a novel, comprehensive telehealth intervention designed to address PPDM using a multifactorial approach [7].

Evaluated in a randomized comparative effectiveness trial with 200 PPDM patients, the comprehensive telehealth program demonstrated better patient outcomes compared to standard telehealth, with greater HbA1c reduction over 12 months (1.59% vs. 0.98%) [8]. In the intervention, patients and staff reported benefits and opportunities for improvements across the following core components of the approach: Diet and Physical Activity, Medication Management, and Depression Support. The study team found high levels of acceptability and described how individual components enhanced diabetes care including providing accountability, reinforcing self-management routines, and active management of medications [9]. Identified opportunities for improvement included tailoring self-management materials to patients’ individual circumstances (e.g., using trauma informed care to support patients with post-traumatic stress disorder) [9]. Due to the effectiveness and patients’ and clinicians’ positive perceptions of the program, there is an opportunity to support translational efforts to implement the approach at scale in real-world practice settings. Thus, using an implementation science theoretical lens, [10, 1] our objective was to extend existing research by identifying and describing implementation barriers and facilitators of the comprehensive telehealth approach with attention across multiple levels that could influence health equity.

## Methods

### Comprehensive telehealth intervention

The project is a secondary analysis of qualitative data collected after the culmination of a 12-month randomized clinical trial (April 2, 2020 to November 2, 2020) [9] during which the comprehensive telehealth intervention was administered to a group (*n* = 101) of participating Veterans with PPDM (defined as ≥ 2 HbA1c values of ≥ 8.5%; without readings < 8.5%; and ≥ 1 appointment with VHA primary care or endocrinology during the prior year). Patients were contacted to participate in the interviews shortly after the completion of the program. The trial was conducted at two VHA medical centers in the Southeastern United States [9]. The comprehensive telehealth program was designed for practical delivery using existing VHA workforce, infrastructure, and resources. Program delivery consisted of five Remote Patient Monitoring-Home Telehealth (RPM-HT) nurse-delivered components delivered across 26 bi-weekly telehealth encounters: telemonitoring, self-management support, diet/activity support, medication management, and depression support. We previously published additional details on the comprehensive telehealth intervention protocol and its clinical delivery (ClinicalTrials.gov Identifier: NCT03520413) [7].

### Study design

We completed a secondary analysis of qualitative data originally collected to assess patient and provider perspectives on the program and identify opportunities to enhance intervention components based on the experience. These qualitative data were re-analyzed with a different analytic goal: To conduct an implementation contextual analysis by identifying barriers and facilitators to supporting the adoption and reach of the comprehensive telehealth approach as part of routine care, outside of the research trial environment.

### Data collection

Qualitative data used in our secondary analysis were collected from diverse, information-rich interviews with patients enrolled in the parent trial who received the comprehensive telehealth intervention, along with frontline clinicians and staff. Staff and clinician interviews were conducted with 3 RPM-HT nurses as well as with 5 administrators/medication managers with first-hand experience delivering and supporting the comprehensive telehealth intervention across both sites. Nineteen patient subjects across both participating clinical sites (Site A *n* = 12; Site B *n* = 7) provided their perspective on the comprehensive telehealth intervention following the 12-month study. The study used a purposive sampling strategy to gather staff and patient participant perspectives from both site locations and patients with varying levels of engagement in the program. Specifically, patient engagement levels used for sampling were based on the number of calls completed with RPM-HT nurses (1–9 calls = low engagement, 10–18 calls = moderate engagement, and ≥ 19 calls = high engagement). Sample size was based on thematic saturation [7].

Qualitative data were collected through phone-based, semi-structured interviews. Participants provided consent to be contacted for these interviews at the time of study enrollment. All interviews were recorded and transcribed by approved staff. The interview guide questions probed participants’ experience, general perceptions of what did and did not work well with the comprehensive telehealth intervention, and impressions of the five intervention components. The clinician and staff interview guide and patient interview guides have been published previously [7].

### Analytic plan

We used the standards for reporting qualitative research (SRQR) checklist to organize and report qualitative data[12]. The completed checklist has been included as Appendix A. Interview transcripts were analyzed using Hamilton’s Rapid Qualitative Analysis methodology, a directed content analysis approach[13, 14]. The data were analyzed and consolidated into matrices by study team members with qualitative research experience (TL, MRE, CD); the team met regularly to ensure rigor of domain definition and application. Qualitative data were managed in Excel-based matrices to facilitate coding management and analysis of patterns. Our team met with a qualitative expert (AS) to discuss methods decisions including: (i) creating an audit trail of relevant project documents and had peer participation in the analytic process to increase study dependability; (ii) conducting analyst triangulation by testing summary templates as a team on Veteran and staff participant transcripts to verify similar content and level of detail; (iii) conducting overreads of a subset of participant summaries[13]; (iv) two team members summarizing each domain based on the study theoretical framework, which were then integrated, increasing both dependability and credibility [15, 16].

The Health Equity Implementation Framework (HEIF) was used as the conceptual framework that guided the qualitative approach [17]. This theoretical framework was selected because it focuses on identifying implementation determinants that could influence health outcomes and disparities at multiple levels including the patients, providers, the clinical encounter, the health system, and larger societal and community contextual factors [18]. These factors include those that are subject to societal or policy-level determinants that have traditionally disadvantaged vulnerable groups like Veterans with PPDM who are more often African American, Hispanic, and unmarried [6]. HEIF has been used to identify implementation determinants of diverse evidence based programs and interventions in VA and non-VA health care settings [19]. The team developed an a priori qualitative codebook guided by HEIF to document definitions, categorizations, and coding decision rules.

## Results

A researcher conducted semi-structured interviews with Veterans across two study sites who received the comprehensive telehealth intervention (*n* = 19). Additional details on Veteran subjects can be found in Table 1. We also interviewed three RPM-HT nurses and five administrators/medication managers across both sites (*n* = 8). Barriers and facilitators to implementation are organized around the HEIF domains and by patient and provider status to facilitate interpretation (See Tables 2 and 3). We identified implementation barriers and facilitators that spanned across HEIF domains.

**Table 1:** Characteristics of veteran interviewees (*n* = 19)

		mean	sd
Age (years)		58.6	8.8
HbA1c (%)*		10.2	1.2
		#	% (rounded)
Sex	Male	15	79%
	Female	4	21%
Race	White	9	47%
	Black	10	53%
Ethnicity	Hispanic/Latino	0	0%
	Not Hispanic/Latino	19	100%
Marital Status	Married	11	58%
	Single, Never Married	2	11%
	Divorced/ Separated/ Widowed	6	32%
Site	Site A	12	63%
	Site B	7	37%
Depression Flag	Yes	7	37%
	No	12	63%
HbA1c Controlled Final Value**	Yes	6	32%
	No	13	68%
Engaged (> or = to 19 calls)	Yes	14	74%
	No	5	26%
**High engagement ≥ 19 Calls**	**Site A**	**7**	**37%**
	**Site B**	**7**	**37%**
Moderate engagement 10–18 Calls	Site B	0	0%
	Site A	3	16%
Low engagement 1–9 Calls	Site B	0	0%
	Site A	2	11%

sd = standard deviation

*Baseline lab value

**Threshold for a controlled HbA1c value in the post period was operationalized as ≤ 8.5%

**Table 2:** HEIF domains and summary of implementation barriers from patient and provider perspectives

HEIF Domain & Description	Barriers	Source	Exemplar Quotations and Findings
*Characteristics of the Innovation* domain includes characteristics related to the behavioral intervention itself, such as its usability (e.g., side effects, modes of delivery), its relative advantage over existing treatments, or its trialability for patients. The “innovation” is the full comprehensive telehealth program protocol which consists of care coordination, telemonitoring, self-management support, diet and physical activity, medication management, and depression support.	Issues with using technology and glucose monitoring equipment	Patient	*“If the VA wants me to send in blood readings*,* or whatever*,* it should be a real simple turn it on*,* send*,* and go.”*
	Frequent medication changes led to greater side effect burden	Patient	At the beginning, (Veteran) was frustrated by all the changes, describing what the doctor did as “shoot and miss”. But as time went on, he got into the structure and the schedule, and needed fewer adjustments.
	Educational content was not always tailored to their needs	Patient	*“The nurse was hyper focused on the diabetes*,* and I just wasn’t on—I guess you’d say I’d suggest making more of a connection between the other issues that the Veteran has (…) and the diabetes.”*
	Opportunity for more involvement of spouses/caregivers	Patient	One patient recommended spouses be included in the program so that they too can understand diabetes management, specifically, “dietary needs”.
	Challenges with setting up Veteran with glucose monitoring equipment	RPM-HT Nurse	*“Pharmacy did not ensure (continuous glucose monitoring device company) reader was working and that patients knew how to use it if it was new to them.”*
	Time-intensive given 1:1 nature of the program and electronic health record documentation burden. May require smaller staffing ratios to avoid understaffing	RPM-HT Nurse	“*Because it takes a lot of time*,* a lot of one-on-one time with the patients. And when you have these full panels*,* you don’t have the time for it. You don’t have the time to invest like we did. You are putting out fires. You’re calling them when their numbers are trending up or trending down. But the servicing that’s going to it*,* you really don’t have time. You really don’t have time to do all the education that we did. We did a lot of decisional education and reinforcement*”
The *Clinical Encounter* domain refers to patient-provider interaction between recipients, which is important to patient satisfaction, trust in providers, and health outcomes. This category includes aspects of the comprehensive telehealth program that are specific to the clinical encounter, such as communication with staff/clinicians.	Poor timing of calls and scheduling created barriers to fitting encounters into existing workflows	RPM-HT Nurse	*“A lot of days*,* I would work later so I could catch them after work. I was trying to catch them on their lunch break*,* which meant*,* my lunch break.”*
	Frustration and potential for side effects when medication adjustments were made without sufficient communication	Patient	Changing insulin via phone was fine, but changing to Glimepride was abrupt for the patient, as there was no warning, it just arrived in his mailbox.
	Training needed to ensure providers are aware of how to motivate diabetes self-management, education, and accessible care pathways to other community and VA programs and resources available	RPM-HT Nurse	*“There is a learning curve to this program that can be challenging at first since other clinical responsibilities are not put on hold.”*
*Patient Factors* is a domain that refers to characteristics or experiences that are specific to the patient and can include beliefs about the telehealth program, an individual’s situation, preferences for communication/engagement, and attitudes towards specific stakeholders or institutions. What about the patients’ situation is going to impact their willingness or ability to engage with the program.	Difficult to adjust to certain lifestyle changes	Patient	“*The problem is that we*,* at least for me*,* with diabetes itself is that I’m a—it’s just a low end area that I’m worried about even if it shouldn’t be. It’s just*,* you know*,* when you’re having a nightmare about something that happened in [place] you can give a [expletive] less if you eat a cookie.”*
	Competing demands and stressors diverted attention away from diabetes self-management	Patient	*“It’s kind of hard to do it [measuring blood sugar] four times a day*,* anyway. Depending on where I am or my schedule.”*
	Success heavily depended on patient availability and engagement level	RPM-HT Nurse	“*if a patient worked or went to school or*,* you know*,* volunteered it became a game of chase sometimes or phone tag a lot*.”
	Emotional distress was a common barrier to patient engagement	RPM-HT Nurse	*“Patients with diabetes can get very defeated.”*
	Difficult to look holistically at the patient’s situation and how that might influence patient engagement	Medication Manager	Information from the chart is incomplete for getting a more holistic picture of patients’ beliefs and motivations for self-care and engagement. Too many phone calls could “backfire” and result in losing buy-in.
The *Provider Factors* domain is specific to the provider or clinician. This can include method of communication, time constraints, or techniques/skills used to respond to care needs or drive engagement to the program.	Coordination between RPM-HT Nurse and Medication Manager within the program could have been better with more structured engagements	Medication Manager	“*What I had*,* uncomfortableness about it I don’t know how else to say it*,* was that I never did get to know the patients myself. And so*,* it seemed*,* you know I had to really trust the nurse to be giving me the correct information*,* and I didn’t even know the patients from the beginning. They were randomly chosen and given to me. So*,* I was offering prescriptions in a box*,* so to speak*,* to somebody I didn’t know. There was a lot of factors like*,* was that person reliably telling us the blood glucose levels? Was the nurse reliably giving me the information correctly from the patient?”*
	There were opportunities to better emphasize the use of motivational interviewing for patients with low adherence	RPM-HT Nurse	*" (…) the nurse in home telehealth alone can’t bear all that. It’s not that we are not equipped or able to do motivational interviewing*,* but (…) we had to do the other questions. However*,* some of them did touch on motivational interviewing in the script*,* it really wasn’t that consistent.”*
*Context* refers to both inner and outer contextual factors. Inner context factors at the local or organizational level can include leadership support for an innovation, feedback processes, the structure of a system, or any formal policies to embed change within a practice. Outer context factors might include incentives or mandates of the larger health care delivery system that patients work within. This includes environmental (in)stability of a political, economic, or cultural nature within the health care system and may relate to engaging with the program or offering it as a program routinely.	COVID-19 made it difficult to attend appointments and engage with program	Patient	Described emotional well-being as ‘not good’, ‘with everything else going on in the world,’ explained emotional wellbeing affected how cared for self and diabetes ‘quite a bit.’
	Distance/geography could prevent engaging with other, related VA services or programs.	Patient	*“While I was in the program. The VA is quite a distance from my house. It’s probably 30 miles from my house. So getting there for studies*,* and plus it’s in [city]*,* south [city]*,* and it’s very crowded down there. So it doesn’t make it easy for me to go. So I probably wouldn’t have gone if they would have ordered me to go. I only need to go periodically when I have to do something. So I didn’t really spend a lot of time with anything outside the diabetes study.”*
	Few opportunities to connect with other Veterans with PPDM	Patient	*“Because the training program*,* the training program talks routinely*,* you know*,* when it comes up*,* when it came up during any given day about if you feel overwhelmed you need to reach out to other Veterans. Well that’s cool if you know who they are. But you—or at least a group. If you know*,* you know*,* here’s a resource. That information wasn’t put out. So if that information exists*,* it should be put out there.”*
	Difficult staying up to date on available VHA programs or resources to refer patients to	Medication Manager	*“VA interventions it’s more kind of sporadic and I come across it more*,* kind of randomly either through reading something or*,* I don’t know*,* just seeing an announcement of some kind. But it’s not like a regular thing (…) that there’s some (…) consistent source that I go to every month (…) to find out what the VA’s implemented.”*
*Societal Influence* are structural social and economic forces that shape implementation. This domain can include transportation, job stability, housing instability, food security, conflict with the criminal justice system, and/or financial barriers.	COVID-19 contributed to poor access to community resources (e.g., recreation areas and gyms)	Patient	*“COVID restricted access to gym facilities*,* making physical activity and exercise goals more difficult to achieve.”*
	COVID-19 created financial and economic disruptions	Patient	*“I’m actually having all my stress right now from dealing with Medicare on my hospital bill.”*
	Stigma related to insulin use in public and management of their diabetes	Patient	*“Full time work and work environment made it difficult to do testing and saw it as a ‘private’ thing that shouldn’t be done in a shared office.”*
	Program did not address unmet social needs (e.g., financial instability, food insecurity or housing instability) that influences diabetes management	RPM-HT Nurse	*“The food resources is a big thing for*,* especially now. I mean it was big before but*,* like I said*,* if you don’t have the resources you need to eat properly for diabetes*,* then you have to eat whatever’s available and a lot of times what’s available is not diabetes friendly.”*
		Patient	*“the nurse was hyper focused on the diabetes*,* and I just wasn’t on—I guess you’d say I’d suggest making more of a connection between the other issues that the Veteran has. (…)You know*,* and the diabetes and make an actual connection between the two would probably be helpful.”*

**Table 3:** HEIF domains and summary of implementation facilitators from patient and provider perspectives

HEIF Domain & Description	Facilitators	Source	Exemplar Quotations and Findings
*Characteristics of the Innovation* domain includes characteristics related to the behavioral intervention itself, such as its usability (e.g., side effects, modes of delivery), its relative advantage over existing treatments, or its trialability for patients. The “innovation” is the full comprehensive telehealth program protocol which consists of care coordination, telemonitoring, self-management support, diet and physical activity, medication management, and depression support.	Program structure, accountability, and requiring participation and collaboration which provided social support	Patient	*“It just gave me the*,* it gave me a lot a structure. And the structure it gave me pretty much gave me self; I don’t want to say self-worth. It made me have more self-confidence with dealing with the diabetes.”*
	Program was built upon established VHA telehealth resources	Medication Manager	*“COVID didn’t impact modality because the program was already virtual care/telehealth; patients also thrived because they already had people/providers checking in with them during the pandemic”*
	Direct and facile lines of communication and continuity with the RPM-HT Nurse.	Patients	*“I received phone calls on a regular*,* every three-day basis to make sure that I was following the instruction*,* taking my medications*,* recording my readings and my tests for glucose. And that’s*,* it was great.”*
	Perception of being a more effective approach	Medication Manager	*“You know maybe perhaps that also because it’s a little bit more comprehensive*,* so the part of the mental health*,* (…) I think that level of detail is perhaps a greater strength. (…) if there’s a more multilayered component where their mental health is also taken into account*,* then that makes it a little bit more of a robust intervention. So I think the fact that it’s more comprehensive and builds on some existing features*,* I think offers promise that it could go further than just what the telehealth program was doing.”*
	Medication management was convenient and seamless	RPM-HT Nurse	*“I had two and they did not hesitate to reach out to me; phone*,* email*,* messenger and extended the same to me if I needed to get in touch with them. And they always made sure to communicate when they weren’t gonna be here and*,* you know*,* who was taking over for them. So that was one of the more seamless parts of the study working with the medication managers.”*
	Multi-disciplinary and team based approach	RPM-HT Nurse	“*We often don’t get that—really teamwork*,* together. We have to piece through and search and search. Where this was really put out saying—This patient has more difficulty*,* so we’re going to add those pieces to it. The mental health is always good*,* but I haven’t had an issue at [site]*,* you know*,* when I need to add mental health. It’s been more hard to get somebody to do consults*,* to get me a dietician*,* to give something*,* or to try to get to the lipid clinic. That’s been more of a challenge. So*,* this program*,* I think*,* showed how much helpful that kind of pulling together as a team could be.*”
The *Clinical Encounter* domain refers to patient-provider interaction between recipients, which is important to patient satisfaction, trust in providers, and health outcomes. This category includes aspects of the comprehensive telehealth program that are specific to the clinical encounter, such as communication with staff/clinicians.	Program was seamless and responsive to care planning / medication changes	Patient	*“Well [name] with the telehealth was very encouraging. You know if she sees a problem where I send in my data she responds very quickly. And helps me get control of it by making suggestions as to what to do between her and the pharmacist that she was working with.”*
	Structure of program created space to discuss clinical and non-clinical needs related to diabetes management	Patient	*“But I think the better part was the support that I had. I knew that I could call and talk to the girls and [name] was just outstanding. (…) Because you know every time*,* I would have a spike*,* and it would really upset me and I knew that I could call [name] or I could call [name] and we would talk about it. You know they were always available for me. And I think that kind of helped me pull out the fact you know*,* you’re not alone and you don’t have to be alone because there are people out there that care.”*
	Nurses were available outside of regular scheduled calls in case issues arose	Patient	*“if I was having any problems they would say call*,* (…). Like if you have any problems call*,* you can call such and such*,* or call us at this number.”*
*Patient Factors* is a domain that refers to characteristics or experiences that are specific to the patient and can include beliefs about the telehealth program, an individual’s situation, preferences for communication/engagement, and attitudes towards specific stakeholders or institutions. What about the patients’ situation is going to impact their willingness or ability to engage with the program.	Complementary to support provided by care partners and family members	Patient	*“So my wife is a big part of my daily routine. She made sure I took my meds right and she’s always helping me out with my meds and the communication between my wife and I and the nurse that was involved*,* like [Name]. Since my wife*,* since she helps me with my meds and everything the communication between her and [Name] was just perfect.”*
	Predictable scheduled engagements were preferred	Patient	*“So when the nurse called me it was*,* first of all*,* she wasn’t consistent as far as every two weeks*,* or at least I don’t recall that was the case.”*
	Improved self-management and other aspect of patient care and/or engagement with other health services	RPM-HT Nurse	“*if you have a patient who’s willing to work with you*,* then you can accomplish anything with the med provider.”*
The *Provider Factors* domain is specific to the provider or clinician. This can include method of communication, time constraints, or techniques/skills used to respond to care needs or drive engagement to the program.	Nurses were trained to provide both clinical (interpreting glucose readings) and non-clinical support (accountability and encouragement)	Patient	*“They’re doing their job*,* but it was—they are like it was more than a job to them (…). Didn’t feel like they were there for a paycheck. Felt like they were there because they really wanted to be there to help somebody.”*
	Empathetic and friendly style of engagement led to positive rapport	Patient	*“I had a good nurse. I mean*,* she stayed on me too. She was really friendly*,* for one. So that also makes you kind of want to do what she wants you to do. I liked—the people I worked with*,* I liked. They had a good team.”*
	Team-based approach fostered collaboration and positively impacted patient communication	Medication Manager	*“To make changes*,* to have that close connection*,* I think was integral in helping make these changes with the Veterans. I think they got a confidence that they didn’t have before*,* that they had that in with that provider. It was very helpful to*,* you know*,* that real-time information*,* sharing back and forth. Instead of where*,* sometimes when you’re in PACT teams*,* you put something out there*,* but you don’t hear anything back*,* or you have to continuously try to find somebody to give you an answer. There was a person that was assigned*,* so to speak*,* to that. And I think that was very helpful.”*
	Nurses helped make connections to other members of the care team as needed (e.g., pharmacist or dietician)	Patient	*“I would say the biggest thing is just knowing that there was another resource. She got me in touch with a dietician to at least answer some questions*,* talk to me about different things. And I think that was very important being able to do that. And knowing that it was there. Same thing with hey your numbers are going up*,* I’m going to have a pharmacist look at it and tell us*,* you know what we think we need to do differently. So those are good things.”*
*Context* refers to both inner and outer contextual factors. Inner context factors at the local or organizational level can include leadership support for an innovation, feedback processes, the structure of a system, or any formal policies to embed change within a practice. Outer context factors might include incentives or mandates of the larger health care delivery system that patients work within. This includes environmental (in)stability of a political, economic, or cultural nature within the health care system and may relate to engaging with the program or offering it as a program routinely.	Veterans felt better equipped to navigate the health system and use telehealth	Patient	*“-it was a combination of the study and the COVID situation that convinced me that I was going to do*,* I was going to try to get on to telehealth as a standard routine.”*
*Societal Influence* are structural social and economic forces that shape implementation. This domain can include transportation, job stability, housing instability, food security, conflict with the criminal justice system, and/or financial barriers.	Participation offered financial benefits in the form of reduced transportation costs	Patient	*“I think it’s great. It saves you gas money*,* keep going to the doctor and also doing your A1C.”*
	Engagement with community of faith provided spiritual support	Patient	*“—for me I needed*,* I sought out some spiritual support because*,* you know*,* changes can be*,* in some respects*,* overwhelming and if you don’t have support group support then it can create a problem. I know that in the program they advocate joining support groups made of up of other diabetic sufferers or folks in diabetic recovery*,* you know*,* to make it less overwhelming.”*
	Reducing transportation barriers to accessing diabetes care improved engagement and, as a result, care quality	Medication Manager	*“And in situations where distance and access to transportation are also a challenge that’s where programs like these can help.”*
	Monetary incentives improved program engagement	RPM HT Nurse	*“As far as what maybe didn’t work*,* and I think we’ll get into that a little further is the—I think that the motivational part of the Veteran coming in*,* because first of all*,* we did choose Veterans for issues. You know*,* these are Veterans that are more resistive*,* for whatever reason. There’s a myriad of reasons they’re more resistive. And I think that in talking with them about motivation*,* a lot of them into the program were right away going—Well*,* I just need the extra money*,* so I’ll just do the—and I did notice that some carried over and continued on after the program was done*,* but some just said*,* you know—When’s my last pay? And then I’m done.”*

### Characteristics of the innovation and clinical encounter

Patients and providers noted that there was opportunity to improve standardization of program educational content regarding physical activity and nutrition, with providers citing this as a barrier to further scaling of the program. Providers reported that the program’s collaborative and multi-disciplinary team approach was a significant facilitator to program success and created structure for team-based care.

Some Veterans felt comprehensive telehealth provided social support through frequent contact with program staff; however, patients also said they would have liked the intervention to provide more emotional support, suggesting the inclusion of a spouse or a caregiver. Although patients liked the telehealth component of the program, some reported experiencing technical difficulties and cited frustration due to frequent and unexpected medication changes. In contrast, providers generally found the program was easy to use, thought that it amplified and complemented existing diabetes care, and believed that it leveraged existing telehealth resources that were able to continue during the pandemic.

Despite liking the format of the program, clinicians found the program to be time-consuming due to its lengthy health record documentation process. Implementation barriers associated with timing of calls and scheduling created barriers to fitting encounters associated with the program into existing workflows, especially when coordinating with primary care. Nurses stated that the program left providers with unpredictable call durations, requiring them to keep flexible call schedules. One respondent said, “*A lot of days*,* I would work later so I could catch them after work. I was trying to catch them on their lunch break*,* which meant*,* my lunch break.”* Providers thought the program needed more staff but noted that the program required a specific skill set, limiting who could be included. One respondent summarized the challenges associated with this part of the program, saying: *“Experience*,* time*,* and training are all barriers to implementation.”* Despite these challenges, providers recognized the program’s strategic value and its alignment with VHA priorities. They saw it as a good use of patient generated data and believed in its potential for long-term sustainability.

### Patient and provider factors

Overall, patients perceived the program to be effective, but that effectiveness depended upon patient engagement as a mechanism to improve diabetes self-management. Patients felt specific stressors, often related to medical, social, and emotional needs, may have influenced their ability to engage with the program and be successful in managing their diabetes. Relatedly, providers noted that engagement (i.e., fidelity or program adherence) with the program was influenced by the holistic situation of the patient.

The establishment of rapport between patients and providers, the program’s telemonitoring feature, and subsequent high engagement with the health care system, were identified as significant facilitators to patient adherence to the program. However, providers also noted that emotional distress due to diabetes management was a common obstacle among patients. Despite this distress, patients expressed satisfaction with the emotional support and encouragement provided by the nurses. Patients described their relationship with their comprehensive telehealth care team, underscoring the importance of continuity of care and rapport.

Patients also thought that nurses’ competencies surrounding promoting accountability for self-management were an important facilitator, with one patient respondent saying, *“If I made a mistake*,* they weren’t hard on me. They’d just say*,* well*,* we want to start from the beginning and get things back on track.”*

Although the program seemed to foster engagement with the health care system, providers noted that higher-touch motivational interviewing and coaching may be necessary for Veterans with lower engagement with the program, with one nurse describing how motivational interviewing with Veterans could be more consistently emphasized.

### Inner & outer context

Both patients and providers thought that COVID-19 was a significant disruptor to diabetes management programs but also noted how the virtual nature of the program helped mitigate transportation and other barriers caused by or exacerbated by the pandemic. One patient discussed how COVID-19 and the virtual component inspired them to consistently utilize telehealth resources: “*In fact I got to tell you this*,* it was—study*,* you know*,* it was a combination of the study and the COVID situation that convinced me that I was going to do*,* I was going to try to get on to telehealth as a standard routine.*” Study staff also noted how the program enabled patients who may have otherwise faced barriers, such as transportation, to be able to access care, with one Medication Manager saying: *“And in situations where distance and access to transportation are also a challenge that’s where programs like these can help.”*

Further, patients appreciated how the program helped them learn how to navigate the health care system and utilize digital resources. Although providers thought workflows and training could have been more standardized, they appreciated how the program was a complementary form of intensified support and not a substitute for primary care. With this, providers also thought the program connected well to existing VHA and community services and resources but recognized that commitment from health system leadership and administrators, alignment with VHA priorities, and buy-in from more primary care providers would be necessary to scale and sustain the program.

### Societal influence

Providers found that program incentives supported uptake and engagement with the program. Although the intervention addresses commonly cited barriers like access, providers thought the program could better address other social needs, like food and housing insecurity, that influence not just engagement with the program but broader goals surrounding diabetes management and clinical outcomes: For example, a nurse highlighted how food insecurity could be influential on patient outcomes in the program either due to availability or cost barriers to healthy eating.

## Discussion

A comprehensive telehealth approach that featured medication management, glucose monitoring, and self-management support was shown to be superior to a standard diabetes telehealth program for patients with PPDM [8]. Prior research has highlighted how this model is acceptable, feasible, and how intervention components directly influence hypothesized mechanisms for improved type 2 diabetes outcomes [9]. The present study built upon this work to identify factors that influence efforts to support implementation of the more intensive, comprehensive approach to managing patients with PPDM despite established care [1]. To do so, we used the HEIF theoretical model to assess implementation determinants that influence health equity. We found important considerations for replicating and scaling the approach with fidelity to how it was tested in the research trial context. These findings have implications for staffing models, workforce development and training protocols, communication channels across multiple disciplines, and careful adaptation of the approach to maintain high levels of dose and navigate potential barriers to patient adherence and engagement.

One key takeaway is that the higher intensity approach introduces multi-level implementation considerations related to ongoing program engagement and fidelity. Our findings related to this phenomenon spanned several HEIF domains since factors that facilitated or hindered program engagement spanned multiple levels of influence. Related to the *Intervention Characteristics* HEIF domain, patients with PPDM respond favorably to the continuity of care over the 26-bi-weekly engagement period and scheduling flexibility to engage patients adequately [9]. *Patient Factors* included the positive response to predictable touch points with members of the care team. However, *Provider Factors* unearthed that this program design feature has implications for the size of a cohort of patients and may introduce challenges given scheduling conflicts and competing clinical demands for providers. Clinical staff highlighted the difficulty of adequately staffing a model like this and the challenge of, even when staff time is dedicated to delivery, making components of the comprehensive approach fit into their existing panels and clinical responsibilities. Predictable protocols may be required for scheduling and completing program encounters and flexibility to adapt program delivery may need to be permissible to overcome potential challenges with program engagement. Finally, at the *Societal Influence* level we found that economic and social factors also contribute to patients’ ongoing engagement and fidelity with the program. Staff and clinicians reported that incentives are valuable to promoting ongoing engagement to the program. Further, responding to unmet social needs, or addressing social and economic factors that may impede patients’ ability to engage consistently with the comprehensive telehealth approach, as part of program participation, could improve program uptake and fidelity. Further, doing so may allow patients to benefit from existing resources to improve social support or food security, among other non-medical drivers of diabetes outcomes [20]. This aligns with other recent qualitative research by Walker et al. (2025) on the important role that social needs play in the context of diabetes management and the potential mechanisms of impact to improve outcomes and equity [21]. In that respect, the VHA is a unique service environment that emphasizes universal screening for unmet social needs and tailored referrals to resources to address co-occurring non-medical needs that may improve self-management behaviors, outcomes, and engagement within the program [22].

Second, integrating comprehensive telehealth programs within the patient centered medical home structure and workflows is perceived as a strength of the approach [9] but may require additional inputs from the primary care team. Providers noted occasional disconnect between primary care providers, nurses, and medication managers. Although patients generally perceived the communication between their providers to be seamless, providers reported that coordination between the various members of the intervention delivery team and the patient’s existing primary care team could be improved. Related to HEIF domains of *Provider Factors* and *Context*, findings suggest that structured communication channels between the program providers and medical home team could promote better coordination, alignment with care planning, and awareness of other programs and resources within the health system that supported PPDM treatment goals.

Third, our findings suggest that scaling the program to serve more patients with PPDM will require careful attention to workforce development and training with attention to aligning with existing systems regarding scope of practice and priorities. Relevant to the *Patient and Provider Factors* domain, providers and staff all highlighted the importance of a specific skillset required to foster shared decision making, accountability, and education. This rapport enabled productive interactions that were thought to contribute to higher levels of patient engagement with the program. These findings also suggest that attention to protocolizing aspects of motivational interviewing and providing sufficient training that is tailored to the goals of the comprehensive telehealth program may be necessary and especially valuable for Veterans with low levels of engagement.

These implementation barriers and facilitators should be interpreted within the broader context of the cost and complexity of the comprehensive telehealth program. Notably, compared to the standard diabetes telehealth approach it was found to be superior to, the comprehensive approach costs an additional $1,519 per patient per year to deliver [9]. Given the potential for scaling, findings from this study may help ‘close the gap’ between the discovery of this effective program and being able to offer the program at scale within routine care settings while retaining the significant benefit observed in a research trial context. This qualitative lens is useful for capturing patient and provider perspectives on the key factors that promote or impede implementation outcomes like adoption, program engagement, and reach.

### Limitations

Despite the strengths of the current study, it is important to acknowledge several limitations. These include a potential lack of transferability beyond the Veteran population that the program was delivered to. Second, the primary qualitative data collection was conducted during the COVID-19 pandemic, which may have influenced perspectives on implementation and impacted the number of interviews that could be conducted. Third, consistent with the secondary analysis study design, the data collection strategy was not aimed exclusively at assessing implementation factors. As a result, it is important to note that additional comprehensive telehealth implementation determinants may exist outside of the comparative effectiveness trial environment that are worthy of future research. Finally, the sampling strategy used to identify staff and patient participants who experienced PPDM (i.e., persistently poor control despite established care) was designed to assess mechanisms for improving outcomes associated with the intervention components, rather than to maximize variation in implementation context for this secondary qualitative analysis. As a result, sampling emphasized levels of program engagement and intervention experience, which may limit the breadth of perspectives on implementation context for the current secondary analyses. Further, despite high levels of racial diversity of the overall study population that matches the diverse population served by the participating health systems, Hispanic patients were underrepresented. These factors may influence the transferability of findings and should be considered when interpreting conclusions related to implementation. Future research should design and test implementation strategies to improve the adoption and reach of this comprehensive telehealth program for those with PPDM [16].

## Conclusion

Comprehensive telehealth approaches improve type 2 diabetes outcomes for those burdened with PPDM, but our findings highlight that implementation requires attention across multiple levels to ensure programs scale equitably and effectively while conveying similar benefits as observed in the research trial context. We found that scheduling, panel management, sustained patient engagement, and a multi-disciplinary team-based approach present unique opportunities and challenges that must be considered to offer a comprehensive telehealth approach at scale. Specifically, these findings have implications for the design of implementation strategies required to adopt, implement, and scale these care models by attending to panel size and scheduling flexibility, establishing team communication channels, integration with existing protocols to address related unmet social needs that impede program uptake and engagement, and workforce development strategies. Future research should test implementation strategies to evaluate their impact on adoption, reach, and fidelity of the approach.

## Data Availability

Data used for this study are available upon reasonable request to the corresponding author.

## Declarations

### Prior presentations

VA Virtual Care Core Cyber Seminar Series, April 9, 2024.

## Abbreviations

HbA1c: Hemoglobin A1c
HEIF: Health Equity Implementation Framework
PPDM: Persistently poorly controlled diabetes mellitus
RPM: HT–Remote Patient Monitoring–Home Telehealth
SRQR: Standards for reporting qualitative research
VHA: Veterans Health Administration
US: United States
